# Anti-inflammatory and antioxidant effects of Tualang honey in alkali injury on the eyes of rabbits: Experimental animal study

**DOI:** 10.1186/1472-6882-11-90

**Published:** 2011-10-09

**Authors:** Karuppannan Bashkaran, Embong Zunaina, Shaharuddin Bakiah, Siti Amrah Sulaiman, KNS Sirajudeen, Venkatesh Naik

**Affiliations:** 1 Department of Ophthalmology, School of Medical Sciences, Health Campus, Universiti Sains Malaysia, Kubang Kerian 16150, Kelantan, Malaysia; 2 Advanced Medical and Dental Institute, Universiti Sains Malaysia, Kepala Batas 13200, Pulau Pinang, Malaysia; 3 Department of Pharmacology, School of Medical Sciences, Health Campus, Universiti Sains Malaysia, Kubang Kerian 16150, Kelantan, Malaysia; 4 Department of Chemical Pathology, School of Medical Sciences, Health Campus, Universiti Sains Malaysia, Kubang Kerian 16150, Kelantan, Malaysia; 5 Department of Pathology, School of Medical Sciences, Health Campus, Universiti Sains Malaysia, Kubang Kerian 16150, Kelantan, Malaysia

## Abstract

**Background:**

Alkali injury is one of the most devastating injuries to the eye. It results in permanent unilateral or bilateral visual impairment. Chemical eye injury is accompanied by an increase in the oxidative stress. Anti-inflammatory and antioxidant agents play a major role in the treatment of chemical eye injuries. The purpose of this study is to evaluate the anti-inflammatory (clinical and histopathological) and antioxidant effects of Tualang honey versus conventional treatment in alkali injury on the eyes of rabbits.

**Methods:**

A preliminary study was carried out prior to the actual study to establish the alkali chemical injury on rabbit's cornea and we found that alkali chemical injury with 2 N NaOH showed severe clinical inflammatory features. In actual study, alkali injury with 2 N NaOH was induced in the right eye of 10 New Zealand White rabbits' cornea. The rabbits were divided into two groups, Group A was given conventional treatment and Group B was treated with both topical and oral Tualang honey. Clinical inflammatory features of the right eye were recorded at 12 hours, 24 hours, 72 hours, 5^th ^day and 7^th ^day post induction of alkali burn on the cornea. The histopathological inflammatory features of the right corneas of all rabbits were also evaluated on day-7. The level of total antioxidant status and lipid peroxidation products in the aqueous humour, vitreous humour and serum at day-7 were estimated biochemically. Fisher's Exact, Chi-Square and Mann-Whitney test were used to analyse the data.

**Results:**

There was no statistically significant difference in clinical inflammatory features (p > 0.05) between honey treated and the conventional treated group at different times of examination. Histopathological examination of the cornea showed the number of polymorphonuclear leucocytes was below 50 for both groups (mild grade). There was also no significant difference in the level of total antioxidant status as well as lipid peroxidation products in aqueous humour (p = 0.117, p = 0.382 respectively), vitreous humour (p = 0.917, p = 0.248 respectively) and serum (p = 0.917, p = 0.332 respectively) between honey treated and the conventional treated group.

**Conclusion:**

Tualang honey has almost the equal effects when compared with the conventional treatment in treating alkali injury on rabbit's eye. Future research with more number of rabbits and control group is warranted to explore the anti-inflammatory and antioxidant effects of Tualang honey.

## Background

Alkali injury of the cornea is one of the most devastating injuries to the eye [1]. The morphological picture after alkali injury could be divided into two phases. In the first phase, there would be infiltrations of the cornea with polymorphonuclear (PMN) leukocytes together with the death of the cellular elements. In this phase, the epithelial cells start to regenerate and epithelization is expected to be completed by the seventh day [2]. In the second phase, there would be a second wave of inflammatory cell infiltration which begins on day 7 and reach the peak between day 14 and 21 in severe injury. The neutrophils release collagenase and their respiratory bursts form superoxide free radical could cause further tissue damage [3,4].

Steroids stabilize the lysosomal vacuoles in leucocytes and prevent the release of proteolytic enzymes which in turn might cause severe secondary complications [5]. Early treatment with corticosteroids or nonsteroidal anti-inflammatory drugs reduces the initial inflammatory cell infiltration as well as the subsequent second-wave infiltration [6].

Chemical injury is one of the causes of oxidative stress in the eye [7]. Free radicals production has been implicated in alkali burns [1]. Free radicals derived from oxygen are known as reactive oxygen species (ROS). Examples of ROS are superoxide anion (O_2_^-^), hydrogen peroxide (H_2_O_2_), hydroxyl radical (OH.), peroxyl radical (ROO), and singlet oxygen (^1^O_2_) [8]. They are mainly produced by leukocytes and by the respiratory mitochondrial chain. They play an important role in cell signaling and defense against bacterial infection.

Lipid peroxidation often occurs in response to oxidative stress [9]. Lipid peroxidation is a process that it causes interaction of oxygen-derived free radicals with polyunsaturated fatty acids [10]. Subsequently the produced electrophilic aldehydes easily get attached covalently to proteins by forming adducts with cysteine, lysine, or histidine residues [10]. Malondialdehyde (MDA) and 4-hydroxynonenal (HNE) are the major products of lipid peroxidation formed [9].

In chemical eye injury, Vitamin C acts as an important antioxidant by reducing peroxides, repairing oxidized biological membrane and neutralizing free radical [11]. Antioxidants prevent disease by scavenging ROS, reducing peroxides, repairing oxidized biological membrane, quenching iron to decrease ROS production and neutralizing ROS via lipid metabolism [11]. The sum of endogenous and food-derived antioxidants represents the total antioxidant activity of the system.

Honey is a popular sweetener and is being used to treat a variety of illness due to its medicinal properties. Honey contains both aqueous and lipophilic antioxidants. These properties enable honey to act at different cellular levels and be an ideal natural antioxidant [12]. Honey with higher water content and with darker colour has more antioxidants. The antioxidants present in honey include Vitamin C, monophenolics, flavonoids, and polyphenolics [13].

Malaysian Tualang honey is a wild multifloral honey. The honey was supplied by Federal Agriculture Marketing Authority (FAMA), Kedah, Malaysia. Tualang honey is produced by *Apis dorsata*, the bees that build their hives on Tualang tree, which was collected from *Koompassia excelsa *(Tualang tree).

Mahaneem Mohamed and co-workers [14] concluded that Tualang honey has a relatively good antioxidant activity due to the good colour intensity and phenolic compounds. Tualang honey has been reported to protect the antioxidant enzymes and decrease the oxidative stress [15]. Honey is also known to have anti-inflammatory properties. Oryan *et al *[16] showed the efficacy of honey in the healing of cutaneous wounds of rabbits on the basis of histopathological and biochemical changes. The honey treated rabbits showed less oedema and necrosis, fewer polymorphonuclear cell infiltration, better wound contraction and improved epithelialization.

The purpose of this study is to evaluate the anti-inflammatory (clinical and histopathological) and antioxidant effects of Tualang honey versus conventional treatment in alkali injury on the eyes of rabbits.

## Methods

### 1. Preliminary Study

A preliminary study was carried out prior to the actual study to establish the alkali chemical injury on rabbit's cornea. Three rabbits were used for the preliminary study.

#### 1.1 Experimental animals

Three New Zealand white adult rabbits (aged 8 - 10 months) weighing between 2.0 and 2.5 kg, with clear cornea were used in this preliminary study. The rabbits were maintained and handled according to the recommendations of the animal ethics guidelines. The animals were housed individually in stainless steel cages under controlled temperature, humidity and 12 hour light: dark cycle. Food (pellet) and water were provided *ad libitum*.

#### 1.2 Induction of alkali injury on cornea

The first rabbit was anaesthetized with an intramuscular dose of Ketamine hydrochloride (Troy Laboratories, New South Wales, Australia) (35 mg/kg of body weight) and Xylazine hydrochloride (Troy Laboratories, New South Wales, Australia) (2.5 mg/kg) approximately 45 minutes prior to induction of alkali injury. The experiment was carried out only on the right eye of each rabbit. A drop of Propacaine hydrochloride 0.5% (Alcaine^®^, Alcon) was instilled to the right eye followed by insertion of barraquer wire speculum. Surplus moisture was removed with cotton tipped applicator.

Filter paper disc (Whatman 3 filter paper) with a 7.5 mm diameter was produced and immersed in 1 N NaOH (Sodium hydroxide) for 30 seconds [4,17]. The alkali soaked filter paper disc was then placed on the central axis of the right cornea, gently held with a forcep for 30 seconds. The cornea then was rinsed with 15 ml of balanced salt solution for 2 minutes. The induction of the alkali chemical injury was done by Investigator A.

The same procedure was repeated for the second rabbit. The size of the filter paper used was 7.5 mm and immersed in 2 N NaOH instead of 1 N NaOH. The duration of filter paper contact on the central axis of the cornea remained at 30 seconds. In the third rabbit, the same concentration of NaOH (2 N NaOH) was used but the duration of filter paper contact on the cornea was increased to 60 seconds.

After induction of the chemical burn, the rabbits were observed individually in their respective cages. Once they were fully conscious, they were observed for signs of stress like inactiveness and reduced food intake. Their weight was monitored daily. They were allowed free mobilization in their cage without any restraint. The right eye was kept open without any pad. There was no treatment given for all the three rabbits.

#### 1.3 Eye examination for clinical inflammatory features

The right eyes of all the rabbits were examined to evaluate the clinical inflammatory features with a binocular loupe at 12, 24 and 72 hours and on the 5^th ^and 7^th ^days post induction of alkali burn [4,18] by the Investigator A. The rabbits were restrained in a wooden box prior to the examination. The eyes were examined for conjunctival hyperemia and corneal edema. Fluorescein strip 1% was used to evaluate the corneal epithelial defect. The grading of the clinical inflammatory features of the cornea was done as per in Table 1. There was no eye discharge noted in all of the three rabbits.

**Table 1:** Grading of the clinical inflammatory features of alkali chemical injury on cornea [4,18].

Clinical features	Grade			
	
	normal	mild	moderate	severe
Conjunctival hyperemia	absent	mild or sectoral engorgement of the conjunctival vessels	diffuse engorgement of the conjunctival vessels	significant engorgement of conjunctival vessels
Corneal edema	absent	present with visible iris details	present without iris details	present without visible pupil
Corneal epithelial defect	absent	defect involving less than one quarter of the corneal surface	defect involving one quarter to one half of the corneal surface	defect involving more than one half of the corneal surface

#### 1.4 Collection of specimens (serum, aqueous humour and vitreous humour) and preparation of corneal button

On day-7, blood sample (about 2 ml) was obtained through marginal ear vein. A 23- gauge needle was used to withdraw the blood. Blood was collected without using an anticoagulant like citrate or heparin [19]. Blood was then subjected to centrifugation at 2000 × g for 15 minutes at 4°C. The top yellow serum layer was pipetted out and freezed at -80°C in a centrifuge tube for the estimation of total antioxidant status and lipid peroxidation products.

The rabbits were then euthanized with intravenous Phenobarbitone sodium (Troy Laboratories, New South Wales, Australia) (125 mg/kg) after the blood collection. Paracentesis was done through the limbal part of the cornea with a sterile 27- gauge needle attached to a 1 ml tuberculin syringe. Aqueous sample of 150 to 200 μl was collected from the right eye [20]. The aqueous sample was transferred to two microcentrifuge tubes. Each tube was labeled accordingly for the estimation of total antioxidant status and lipid peroxidation products. The samples were stored frozen at -80°C.

Following that, the right eye was fixated with the conjunctival forcep and the anterior chamber was entered at the limbus with a scalpel blade. The cornea was excised with corneal scissors, placed in 10% formalin and sent for histopathological examination.

The vitreous samples were aspirated with 5 ml syringe without needle after harvesting the cornea. Vitreous sample of about 0.3 ml (300 μl) was withdrawn carefully from the right eye. Twenty (20) μl and 100 μl of the sample were used for the estimation of total antioxidant status and lipid peroxidation products respectively. Each sample was transferred to a 2 ml microcentrifuge tube and stored frozen at -40°C prior to spectrophotometric analysis.

After completing the collection of specimens (serum, aqueous humour, vitreous humour and cornea), the rabbits were then disposed via biohazard bags.

#### 1.5 Light-microscopic examination of the cornea for histopathological inflammatory features

The excised corneas were prepared for light microscopic examination. The corneas were fixed with 10% formaldehyde and dehydrated in a series of alcohol concentration. The cornea tissues were treated with xylene to clear the alcohol and then impregnated in paraffin. The required tissue was sliced with a microtome blade with a section thickness set at 3-5 micron. Then it was stained with haematoxylin and eosin for light-microscopic examination.

Light-microscopic quantitation was performed by counting PMN leukocytes in tissue sections per high-power field (x40 objective, x10 ocular) with a binocular microscope. Initially the slide was seen under low power 10 × field for most populated area with inflammatory cells. Then counting of the neutrophils was done under 400 × field in three adjacent areas. The average number of counting PMN was taken and graded as follows: 1-50 = mild, 51-100 = moderate, 101-200 = severe and 201 or above = very severe, modified from the study of Öztürk [4]. All the histopathological examination of the cornea was done by Investigator B.

#### 1.6 Measurement of total antioxidant status (TAS) and lipid peroxidation products

Total antioxidant activity was measured by Cayman Total antioxidant assay kit (Cayman, MI, USA). The principle is based on the antioxidant potential of pure compounds and biological fluids to both quench and inhibit the formation of a colored radical cation produced by the action of metmyoglobin and hydrogen peroxide on 2, 2'-azino-*bis*-3-ethylbenzothiazoline-6-sulfonic acid (ABTS) [21].

Lipid peroxidation products were measured by Cayman TBARS assay kit (Cayman, MI, USA). The most widely used method is the reaction of MDA with Thiobarbituricacid (TBA) as used in this study. It is dependent on the formation of a red pigment that results from the reaction of TBA with oxidized lipids.

Both the tests were carried out according to the Cayman's kit protocol. Ten (10) μl and 100 μl of the samples were used for the estimation of TAS and lipid peroxidation products respectively. All the measurement of biochemical parameters was done by Investigator C.

#### 1.7 Results of the preliminary study

**Figure 1:**
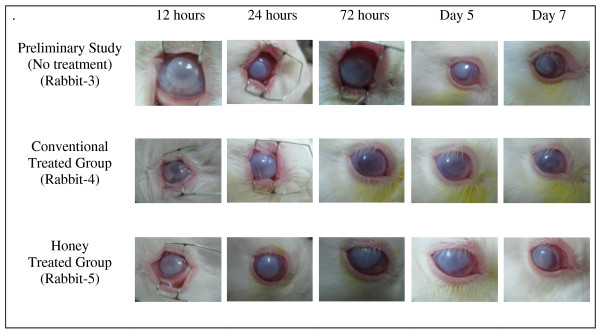
**Conjunctival hyperemia and corneal edema post induction of alkali injury on rabbit's cornea**.

**Figure 2:**
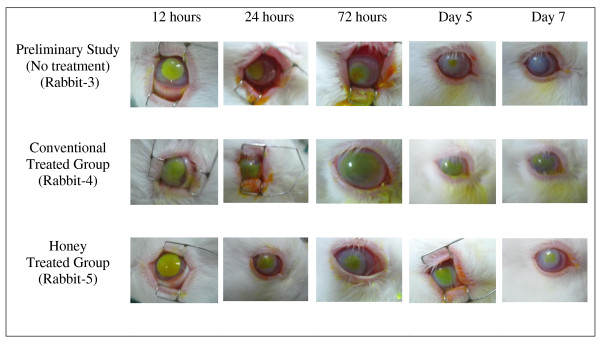
**Corneal epithelial defect post induction of alkali injury on rabbit's cornea**.

**Figure 3:**
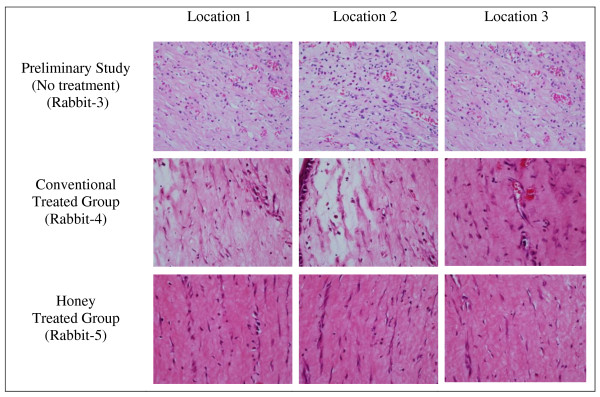
**Histopathological inflammatory features on day 7 post induction of alkali injury on rabbit's cornea**.

The result of the preliminary study for clinical inflammatory and histopathological inflammatory features of alkali injury on rabbit's cornea is shown in Table 2. Rabbit-3 shows severe clinical inflammatory features in terms of conjunctival hyperemia (Figure 1), corneal edema (Figure 1) and corneal epithelial defect (Figure 2). Light-microscopic examination of the cornea found that the number of PMN leucocytes was below 50 (mild grade) for rabbit-2 and rabbit-3 and absent in rabbit-1 (Table 2). Figure 3 shows the histopathological inflammatory features of the rabbit-3 in three adjacent areas.

**Table 2:** Results of the preliminary study in alkali chemical injury on rabbit's cornea

Rabbit	NaOH (Concentration and duration of exposure)	Clinical inflammatory features (on day-7)			Histopathological inflammatory features (on day-7)
		
		Conjunctival hyperemia	Corneal edema	Corneal epithelial defect	Grading of PMN
1	1N NaOH (30 seconds)	moderate	moderate	mild	Nil
2	2N NaOH (30 seconds)	moderate	moderate	mild	mild
3	2N NaOH (60 seconds)	severe	severe	severe	mild

N: Normality, NaOH: Sodium hydroxide, PMN:polymorphonuclear

In the preliminary study, the samples from aqueous humour, vitreous humour and serum was used to optimize the absorbance value within the range of the standards for the estimation of total antioxidant status and lipid peroxidation products. We observed that 10 times dilution of the samples (aqueous humour and vitreous humour) gave the optimum results in which the absorbance value of the samples showed absorbance within the range of the standards. The serum however was diluted 20 times as per mentioned in the catalogue and showed absorbance within the range of the standards.

For the estimation of lipid peroxidation products, the samples without dilution gave the optimum results and showed absorbance within the range of the standards.

### 2. Actual Study

#### 2.1 Experimental animals

Actual study was conducted with 5 rabbits in each group namely Group A - conventional treated group and Group B - honey treated group. Ten New Zealand white adult rabbits (aged 8 - 10 months) weighing between 2.0 and 2.5 kg, with clear cornea were used in this actual study. The rabbits were maintained as per mentioned in 1.1.

#### 2.2 Induction of alkali injury on cornea

Alkali chemical injury was induced as per mentioned in 1.2. The third rabbit in the preliminary study showed severe clinical inflammatory features. Thus the method of induction of alkali chemical injury with 2 N NaOH and with corneal contact of 60 seconds was chosen in this actual study. The animals were cared and observed as per mentioned in 1.2.

#### 2.3 Treatment groups

The rabbits were divided into 2 groups (Group A and Group B) consisting of 5 rabbits in each group by using double block randomization. Group A was the conventional treated group, treated with one drop of topical Prednisolone acetate (Predforte^®^, Allergen) 1% four times/day and one drop of topical Ciprofloxacin 0.3% (Cipmax^®^, Alcon) four times/day along with oral administration of ascorbic acid (0.6 gm/kg/day) for 1 week [22]. Group B was the honey treated group, treated with one drop of topical Tualang honey and diluted in normal saline (30%) applied for four times/day along with oral administration of Tualang honey (1.0 gm/kg/day) for 1 week [15].

Tualang honey and ascorbic acid were supplemented orally by means of 1 ml or 3 ml syringe in the morning. The needed ascorbic acid (powder form) was measured with a weighing scale and mixed with distilled water to dissolve it prior to feeding. Thirty percent honey, for topical application was freshly prepared everyday by mixing 1.5 ml of honey with 3.5 ml of distilled water in the morning just before the first instillation. Honey was diluted to reduce its viscosity. Following that, the prepared honey was kept in the refrigerator for the next 3 instillations for the same day.

Tualang honey used in this study was subjected to gamma irradiation at 25 kGy at Sterilgamma (M) Sdn. Bhd. (Selangor, Malaysia) for the purpose of sterilization. This radiation was shown not to cause significant loss of antibacterial activity and there was no viable clostridial spores after the process [23].

#### 2.4 Eye examination for clinical inflammatory features

The right eyes of all the rabbits were examined to evaluate the clinical inflammatory features with a binocular loupe at 12, 24 and 72 hours and on the 5^th ^and 7^th ^days post induction of alkali burn as per mentioned in 1.3 by the Investigator A. There was no eye discharge noted in all of the rabbits in the honey treated group as well as in the conventional treated group.

#### 2.5 Collection of specimens (serum, aqueous humour and vitreous humour) and preparation of corneal button

The collection of serum, aqueous humour and vitreous humour (from the right eye) as well as preparation of the corneal button of the right eye were done on the 7^th ^days post induction of alkali burn as per mentioned in 1.4. After completing the collection of specimens (serum, aqueous humour, vitreous humour and cornea), the rabbits were then disposed via biohazard bags.

#### 2.6 Light-microscopic examination of the cornea for histopathological inflammatory features

The excised corneas were prepared for light microscopic examination and the grading of the histopathological inflammatory features was evaluated as per mentioned in 1.5.

#### 2.7 Measurement of total antioxidant status (TAS) and lipid peroxidation products

##### Measurement of the total antioxidant status

The measurement of the total antioxidant status was done as per mentioned in 1.6. In the preliminary study, the sample that was diluted ten times showed absorbance within the range of the standards. Thus the sample dilution factor used was 10 times in this actual study in order to bring the antioxidants level within the standard curve. The average absorbance of standards was plotted as a function of the final Trolox concentration (mM) (Cayman's kit protocol).

#### 

##### Measurement of lipid peroxidation products

The measurement of the lipid peroxidation products was done as per mentioned in 1.6. Corrected absorbance was determined by subtracting the absorbance of the standard A (O μM) from itself and all other values (both standards and samples). The absorbance values of each standard were plotted as a function of MDA concentration (Cayman's kit protocol).

### 3. Ethical approval

This study was appproved by the Research and Ethical Committee, Health Campus, Universiti Sains Malaysia on 16^th ^March 2009 and also by Animal Ethics Committee, Health Campus, Universiti Sains Malaysia on 7^th ^May 2009.

### 4. Statistical Analysis

The data collected were analyzed using Statistical Package for Social Science (SPSS) software version 12.1. Fisher Exact test and Chi square test were used to analyze the results where appropriate. For the data that was not normally distributed, Mann-Whitney test was used for the statistical analysis. Thus the values are expressed as median (interquartile range). The p value of < 0.05 is considered as statistically significant.

## Results

### Clinical anti-inflammatory effect

The association between grading of conjunctival hyperemia and treatment type in alkali chemical injury on the eyes of rabbits is shown in Table 3. The conjunctival hyperemia noted to be severe in both groups till 72 hours post induction of alkali injury. However one rabbit in the conventional treated group showed moderate severity at 72 hours. On the 7^th ^day of examination, almost all the rabbits improved to moderate category of conjunctival hyperemia except two rabbits, one each from both groups. Figure 1 shows the features of conjunctival hyperemia in conventional treated group (rabbit-4) and in honey treated group (rabbit-5).

**Table 3:** Clinical inflammatory features of alkali chemical injury on rabbit's cornea between conventional treated and honey treated group

	Conventional Treated Group n = 5				Honey Treated Group n = 5		
	Mild	Moderate	Severe	Mild	Moderate	Severe	P value
**Conjunctival hyperemia**							
12 hours	0	0	5	0	0	5	-
24 hours	0	0	5	0	0	5	-
72 hours	0	1	4	0	0	5	1.000^a^
5 days	0	4	1	0	2	3	0.524^a^
7 days	0	4	1	0	4	1	1.000^a^

**Corneal edema**							
12 hours	0	2	3	0	2	3	1.000^a^
24 hours	0	2	3	0	0	5	0.444^a^
72 hours	1	1	3	0	0	5	0.287^b^
5 days	1	0	4	0	0	5	1.000^a^
7 days	1	0	4	0	1	4	0.368^b^

**Epithelial defect**							
12 hours	0	0	5	0	0	5	-
24 hours	0	0	5	0	0	5	-
72 hours	0	0	5	0	2	3	0.444^a^
5 days	0	1	4	1	1	3	0.565^b^
7 days	0	4	1	3	2	0	0.097^b^

^a^Fisher Exact test, p < 0.05 significant

^b^Chi-Square test, p < 0.05 significant

There was no statistically significant difference in the grading of conjunctival hyperemia between honey treated and conventional treated group at 72 hours (p = 1.000), 5^th ^day (p = 0.524), and 7^th ^day (p = 1.000) of clinical examination. Statistical analysis was not done for the 12 hours and 24 hours of clinical examination (both groups) as all the rabbits fell under severe category.

The association between grading of corneal edema and treatment type in alkali chemical injury on the eyes of rabbits is shown in Table 3. Only one rabbit in the honey treated group showed improvement to moderate category of corneal edema on the 7^th ^day of examination. One rabbit in the conventional group improved to mild category at 72 hours itself and remained the same till the 7^th ^day. The corneal edema was severe in the rest of the rabbits on day-7. Figure 1 shows the features of corneal edema in conventional treated group (rabbit-4) and in honey treated group (rabbit-5).

There was no statistically significant difference in the grading of corneal edema between honey treated and conventional treated group at different times of clinical examination (12 hours, p = 1.000; 24 hours, p = 0.444; 72 hours, p = 0.287; 5 days, p = 1.000; 7 days, p = 0.368).

The association between grading of corneal epithelial defect and treatment type in alkali chemical injury on the eyes of rabbits is shown in Table 3. Two rabbits in the honey treated group showed improvement to moderate category of corneal epithelial defect at 72 hours whereas all the rabbits in the conventional treated group remained in the severe category. On the 7^th ^day, 4 out of 5 rabbits in the conventional treated group improved to moderate category. The honey treated group sustained only mild epithelial defect in 3 rabbits and the rest were in moderate category. Figure 2 shows the features of corneal epithelial defect in conventional treated group (rabbit-4) and in honey treated group (rabbit-5).

Clinically, the honey treated group showed better improvement in the healing of the corneal epithelial defect compared to the conventional treated group. However there was no statistically significant difference in the grading of corneal epithelial defect between honey treated and conventional treated group at 72 hours (p = 0.444), 5^th ^day (p = 0.565) and 7^th ^day (p = 0.097) of clinical examination. Statistical analysis was not done for the 12 hours and 24 hours of clinical examination as all the rabbits (both groups) fell under severe category.

### Histopathological anti-inflammatory effect

Light-microscopic examination of the cornea found that the number of PMN leucocytes was below 50 for both groups (mild grade). Statistical analysis was not done as all the rabbits (both groups) fell under mild category. Figure 3 shows the features of histopathological inflammatory features in conventional treated group (rabbit-4) and in honey treated group (rabbit-5).

### Anti-oxidant effect

The honey treated group has a higher mean level of total antioxidant in aqueous humour (3.19 ± 1. 82 mM) and serum (14.97 ± 0.71 mM) when compared with that of the conventional group (1.74 ± 0.68 mM, 14.77 ± 1.57 mM respectively). However the mean level of total antioxidant in vitreous humour is less in the honey treated group (honey treated group: 2.04 ± 0.50 mM, conventional treated group: 2.57 ± 1.85 mM). The comparison between the levels of total antioxidant status in aqueous humour, vitreous humour and serum in alkali chemical injury on the eyes of rabbits between honey treated and the conventional treated group is shown in Table 4.

**Table 4:** Level of anti oxidant status and lipid peroxidation product in alkali chemical injury on rabbit's cornea between conventional treated and honey treated groups

	Conventional Treated Group	Honey Treated Group	P value
**Anti oxidant status (mM)**			
**Aqueous**			
mean (SD)	1.74 (0.68)	3.19 (1.82)	
median (IQR)	1.55 (1.07)	2.13 (3.31)	0.117
**Vitreous**			
mean (SD)	2.57 (1.85)	2.04 (0.50)	
median (IQR)	1.85 (2.72)	2.05 (0.93)	0.917
**Serum**			
mean (SD)	14.77 (1.57)	14.97 (0.71)	
median (IQR)	14.71 (3.01)	15.07 (1.04)	0.917

**Lipid peroxidation product (μM)**			
**Aqueous**			
mean (SD)	1.59 (1.05)	1.12 (1.15)	
median (IQR)	1.47 (2.06)	0.29 (2.06)	0.382
**Vitreous**			
mean (SD)	4.18 (2.99)	2.29 (0.89)	
median (IQR)	3.82 (5.59)	2.06 (1.76)	0.248
**Serum**			
mean (SD)	7.47 (2.92)	5.59 (1.44)	
median (IQR)	6.18 (3.82)	6.18 (2.65)	0.332

Mann-Whitney test, p < 0.05 significant

The honey treated group has a lower mean level of lipid peroxidation products in aqueous humour, vitreous humour and serum (1.12 ± 1.15 μm, 2.29 ± 0.89 μm, 5.59 ± 1.44 μm respectively) when compared with that of the conventional group (1.59 ± 1.05 μm, 4.18 ± 2.99 μm, 7.47 ± 2.92 μm respectively) The comparison between the levels of lipid peroxidation products in aqueous humour, vitreous humour and serum in alkali chemical injury on the eyes of rabbits between honey treated and the conventional treated group is shown in Table 4.

Mann-Whitney test was used for the statistical analysis as the data was not normally distributed. Thus the values are expressed as median (interquartile range). There was no significant difference in the median level of total antioxidant status as well as lipid peroxidation products in aqueous humour (p = 0.117, p = 0.382 respectively), vitreous humour (p = 0.917, p = 0.248 respectively) and serum (p = 0.917, p = 0.332 respectively) between honey treated and the conventional treated group.

## Discussion

Chemical eye injury is accompanied by decrease in the antioxidant protection system as well as activation of the free radical lipid peroxidation. To the best of our knowledge, no studies have been done on the effect of Malaysian honey on the eye.

In this study, the honey treatment is noted to be as good as the conventional treatment in treating the conjunctival hyperemia, corneal edema and epithelial healing due to alkali chemical injury. In a study by Alio *et al *[24] antioxidant topical treatment with Dimethythiourea has been found to be efficient in reducing inflammatory reactions during acute corneal inflammation.

Hallberg and his colleagues [25] suggested that free radicals delay the corneal epithelial wound healing in rabbits. This was evidenced by improvement in the epithelial defect in diabetic rats treated with Trolox antioxidant when compared with that of untreated diabetic rats. However the corneal epithelial wound induced was only 3 mm. This current study revealed that the honey treated group is as good as the conventional treated group in the healing process of corneal epithelial defect.

In the histolopathological evaluation, there was only mild corneal infiltration by PMN in all the rabbits. This shows that the honey treatment has the anti-inflammatory component comparable to that of the conventional treatment. Our results are supported by the study by Öztürk *et al *[4]. In that study the degree of inflammatory cell infiltration was significantly lower in the group treated with topical dexamethasone and also the one treated with Propolis. However the strength of the NaOH used was 1 N and the exposure time was only 30 seconds. They also noted no statistically significant difference between those two groups as in this study. As a conclusion, they agreed that Propolis has an anti-inflammatory component comparable to dexamethasone in chemical corneal injury.

Study by Pfister *et al *[26] demonstrated that daily subcutaneous injection of ascorbic acid had significantly raised the plasma ascorbic acid level greater than that of the control. Moreover the level of ascorbic acid in the aqueous humour has increased as well. However the groups treated with topical ascorbic acid showed higher levels of ascorbic acid. This explains that the transport of parenteral ascorbic acid had slower access to cross the blood-aqueous barrier.

Pfister's study showed increment in the mean level of ascorbic acid in the aqueous humour with 35 seconds of chemical burn exposure with 1 N NaOH [27]. However the increment in the ascorbic acid level was noted to be less than his previous study in 1977 where the chemical injury was induced with an exposure of 20 seconds [27]. The characteristic of antioxidant activity of ascorbic acid is measured as total antioxidant status.

In this current study, the concentration of NaOH used was 2 N and the exposure time was 60 seconds. The concentration of total antioxidant status was noted to be higher in the honey treated group when compared with the conventional treated group in the aqueous humour and serum. However the total antioxidant status in the vitreous humour was lower in the honey treated group. But those results were statistically not significant.

Total antioxidant activity in the aqueous humour, vitreous humour and retina was noted to be higher in the groups treated with oral antioxidants and topical anti-inflammatory drugs when compared with the control group where only topical anti-inflammatory drugs were given. This denotes that oral supplement can increase the antioxidant capacity in eye tissues of rabbits [28].

In the same study, the concentration of MDA in the aqueous humour, vitreous humour and retina has also been explored. As expected, the MDA concentration was lower in the groups treated with oral antioxidants, post induction of alkali chemical injury [28]. The concentration of MDA and total antioxidant status were inversely related i.e. if the concentration of the MDA is high, the total antioxidant status will be less and vice versa.

In this current study, the concentration of MDA was inversely related when compared between the two groups. For example if the total antioxidant status of aqueous humour is more in the honey treated group, the concentration of MDA will be less in this group when compared with that of the conventional group. The lower value of lipid peroxidation products in honey treated group clearly denotes the protective role of honey as an antioxidant against the oxidative stress induced by the alkali chemical injury. This correlates with the study by Gakhramanov [28]. However the method of chemical eye injury induced in that study was not mentioned.

Schramm and colleagues [13] have concluded that oral administration of honey can increase the plasma antioxidant level. In their study, honey fed at 1.5 gm/kg body weight found to raise the plasma antioxidant level. Oxidative stress was not induced in their study. In this current study, the honey treated group showed higher plasma antioxidant level than the conventional group. However the dose was only 1.0 gm/kg and the oxidative stress was induced by means of alkali chemical injury.

In this study, we found that there was no clinical sign of infection with the absence of eye discharge in all of the eyes with alkali chemical injury in honey treated group as well as in conventional treated group. Although honey has a high content of sugar but it has a low content of water, together with acidity property [29]. These characteristics will prevent microbial growth. Honey also will generate hydrogen peroxide when diluted [30,31] and this hydrogen peroxide is the major contributor to the antimicrobial activity [30,32].

## Conclusions

Tualang honey has almost the equal effects when compared with the conventional treatment in treating alkali chemical injury on rabbit's eye. Future research with more number of rabbits and control group is warranted to explore the anti-inflammatory and antioxidant effects of Tualang honey.

## Competing interests

The authors declare that they have no competing interests.

## Authors' contributions

KB carried out the induction of chemical burn and clinical examination, performed the analysis of data, interpretation of results and drafted the manuscript. EZ participated in the design of the study, edited the manuscript, involved in the acquisition of the grant and monitored the research. SB participated in the design of the study. SAS was involved in the preparation of honey, design of the study and revised the manuscript for the intellectual content. KNSS carried out the biochemical parameter test. VN performed the histopathological examination. All authors have read and approved the final manuscript.

## Pre-publication history

The pre-publication history for this paper can be accessed here:

http://www.biomedcentral.com/1472-6882/11/90/prepub
